# Fixed Air in Tinea Capitis

**Published:** 1809-05

**Authors:** 


### *To the Editors of the Medical and Phyſical Journal.*

Gentlemen,

In your very valuable Miscellany for the last month, I find some inquiries made after a remedy for the disease in the hair of children, usually called ring worms, by Mr. James Hall. I cannot give him the information he requires about the predisposing cause, or the best way of preventing its spreading in large families, otherways than separation, for I do believe it to be infectious; but I can, with confidence, recommend to him to try fixed air, extracted from marble dust by the vitriolic acid; I have found it to answer every expectation, and to give me the utmost satisfaction. The apparatus for applying it, is a bladder cut at its fundus, in form like a night cap, which is to be fixed close at its edge, air tight, by a bandage round the head, the other part of the bladder being loose to contain the fixed air; it may be filled from a quart bottle, by means of a tube made of a hog's gut, one end being fixed to the bottle, and the other end to the neck of the bladder, which should be pervious for the admission of the fixed air. The ingredients are water one pint, vitriolic acid one ounce, and marble dust two ounces; it must be applied at least twice a day, a quarter of an hour each time; and I can venture to say, that if Mr. H. will give it a fair trial, he will find it as deserving of his confidence, as it has done that of

*March* 5, 1809. Yours, &c. W. D.

